# Oxiracetam and physical activity in preventing cognitive decline after stroke: A multicenter, randomized controlled trial

**DOI:** 10.1093/esj/23969873251350141

**Published:** 2026-01-01

**Authors:** Jae-Sung Lim, Joung-Ho Rha, Jong-Ho Park, Kyungbok Lee, Dae-Il Chang, Sung Hyuk Heo, Yeong Bae Lee, Jee-Hyun Kwon, Eung-Gyu Kim, Jay Chol Choi, Man-Seok Park, Kyung-Hee Cho, Jae-Kwan Cha, Mi Sun Oh, Byung-Chul Lee, Hahn Young Kim, Kyungmi Oh, Hyun-Young Park, Sanghak Yi, Tai Hwan Park, Jae-Hyeok Heo, Keun-Hwa Jung, Chulho Kim, Soo Joo Lee, Jae Guk Kim, Dong-Eog Kim, Jong-Moo Park, Kyusik Kang, Jun Hong Lee, Jong-Won Chung, Kwang-Yeol Park, Won-Jin Moon, Hyuntae Park, Seongryu Bae, Yeonwook Kang, Hannah Jung, Juneyoung Lee, Hee-Joon Bae

**Affiliations:** Department of Neurology, Asan Medical Center, University of Ulsan College of Medicine, Seoul, Republic of Korea; Department of Neurology, Inha University Hospital, Inha University School of Medicine, Incheon, Republic of Korea; Department of Neurology, Eunpyeong St. Mary’s Hospital, The Catholic University of Korea, Seoul, Republic of Korea; Department of Neurology, Samsung Changwon Hospital, Sungkyunkwan University School of Medicine, Changwon, Republic of Korea; Department of Neurology, Soonchunhyang University Seoul Hospital, Soonchunhyang University College of Medicine, Seoul, Republic of Korea; Department of Neurology, Kyung Hee University Hospital, Kyung Hee University College of Medicine, Seoul, Republic of Korea; Department of Neurology, Gil Medical Center, Gachon University College of Medicine, Incheon, Republic of Korea; Department of Neurology, Kyung Hee University Hospital, Kyung Hee University College of Medicine, Seoul, Republic of Korea; Department of Neurology, Gil Medical Center, Gachon University College of Medicine, Incheon, Republic of Korea; Department of Neurology, Ulsan University Hospital, University of Ulsan College of Medicine, Ulsan, Republic of Korea; Department of Neurology, Inje University Busan Paik Hospital, Inje University College of Medicine, Busan, Republic of Korea; Department of Neurology, Jeju National University Hospital, Jeju National University College of Medicine, Jeju, Republic of Korea; Department of Neurology, Chonnam National University Hospital, Chonnam National University Medical School, Gwangju, Republic of Korea; Department of Neurology, Korea University Anam Hospital, Korea University College of Medicine, Seoul, Republic of Korea; Department of Neurology, Dong-A University Hospital, Dong-A University College of Medicine, Busan, Republic of Korea; Department of Neurology, Hallym University Sacred Heart Hospital, Hallym University College of Medicine, Anyang, Republic of Korea; Department of Neurology, Hallym University Sacred Heart Hospital, Hallym University College of Medicine, Anyang, Republic of Korea; Department of Neurology, Konkuk University Medical Center, Konkuk University College of Medicine, Seoul, Republic of Korea; Department of Neurology, Korea Univeristy Guro Hospital, Korea University College of Medicine, Seoul, Republic of Korea; Department of Neurology, Wonkwang University Hospital, Wonkwang University College of Medicine, Iksan, Republic of Korea; Department of Neurology, Wonkwang University Hospital, Wonkwang University College of Medicine, Iksan, Republic of Korea; Department of Neurology, Seoul Medical Center, Seoul, Republic of Korea; Department of Neurology, Seoul Medical Center, Seoul, Republic of Korea; Department of Neurology, Seoul National University Hospital, Seoul National University College of Medicine, Seoul, Republic of Korea; Department of Neurology, Hallym University Chuncheon Sacred Heart Hospital, Hallym University College of Medicine, Chuncheon, Republic of Korea; Department of Neurology, Daejeon Eulji Medical Center, Eulji University School of Medicine, Daejeon, Republic of Korea; Department of Neurology, Daejeon Eulji Medical Center, Eulji University School of Medicine, Daejeon, Republic of Korea; Department of Neurology, Dongguk University Ilsan Hospital, Dongguk University College of Medicine, Goyang, Republic of Korea; Department of Neurology, Uijeongbu Eulji Medical Center, Eulji University School of Medicine, Uijeongbu, Republic of Korea; Department of Neurology, Nowon Eulji Medical Center, Eulji University School of Medicine, Seoul, Republic of Korea; Department of Neurology, National Health Insurance Service Ilsan Hospital, Goyang, Republic of Korea; Department of Neurology, Samsung Medical Center, Sungkyunkwan University School of Medicine, Seoul, Republic of Korea; Department of Neurology, Chung-Ang University Medical Center, Chung-Ang University College of Medicine, Seoul, Republic of Korea; Department of Radiology, Konkuk University Medical Center, Konkuk University College of Medicine, Seoul, Republic of Korea; Department of Healthcare, Dong-A University, Dong-A University College of Medicine, Busan, Republic of Korea; Department of Healthcare, Dong-A University, Dong-A University College of Medicine, Busan, Republic of Korea; Department of Psychology, Hallym University, Chuncheon, Republic of Korea; Department of Biostatistics, Korea University, Seoul, Republic of Korea; Department of Biostatistics, Korea University, Seoul, Republic of Korea; Department of Neurology, Seoul National University Bundang Hospital, Seoul National University College of Medicine, Seongnam, Republic of Korea

**Keywords:** Vascular dementia, oxiracetam, clinical trials, physical activity, exercise

## Abstract

**Introduction:**

This multicenter, double-blind, placebo-controlled trial, commissioned by South Korea’s Ministry of Food and Drug Safety, evaluated the effect of oxiracetam for preventing post-stroke cognitive impairment (PSCI) and explored potential interaction with physical activity using neuroimaging.

**Patients and methods:**

Patients at high risk of PSCI, reporting subjective cognitive decline ⩾3 months after stroke, were randomized 1:1 to receive oxiracetam or placebo for 36 weeks. Physical activity was tracked via wrist-worn actigraphy. Coprimary endpoints were changes in Mini-Mental State Examination (MMSE) and Clinical Dementia Rating–Sum of Boxes (CDR-SB). Secondary outcomes included neuropsychological assessments and resting-state functional magnetic resonance imaging network metrics.

**Results:**

Of 500 enrolled participants (mean age 68.9 years; median 32 months post-stroke), 457 completed the study. There were no statistically significant differences between groups in changes in MMSE (oxiracetam: +0.13 ± 2.27 vs placebo: +0.27 ± 2.09; *p* = 0.49) or CDR-SB scores (–0.14 ± 0.70 vs −0.08 ± 0.80; *p* = 0.38). No evidence of interaction was observed between oxiracetam and physical activity. Exploratory analyses suggested favorable trends in functional segregation and CDR-SB scores among highly active oxiracetam participants.

**Discussion and conclusion:**

Oxiracetam did not demonstrate benefit in preventing PSCI in high-risk patients. These findings support the recent regulatory decision to suspend its use in South Korea.

## Graphical Abstract

**Graphical abstract:**
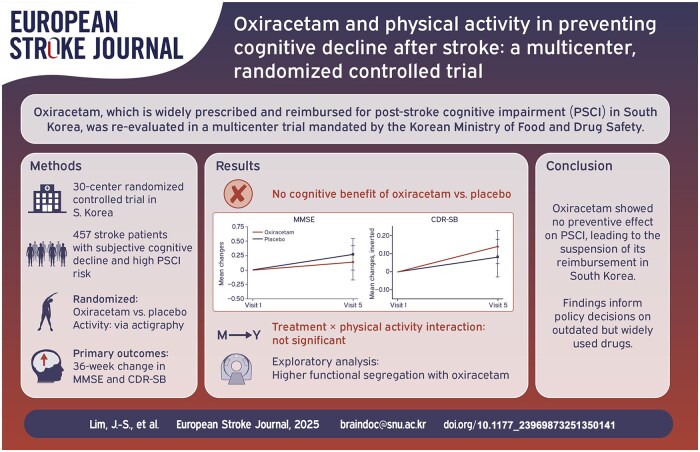

## Introduction

Post-stroke cognitive impairment (PSCI) is a common consequence of stroke, impairing functional recovery and long-term quality of life.^1^ Despite its clinical relevance, effective pharmacologic treatments remain limited. In South Korea, nootropics such as oxiracetam are widely prescribed and reimbursed, with annual expenditures reaching 22.8 billion KRW (€17 million) in 2021.^2,3^ However, supporting evidence on its efficacy is largely outdated and inconclusive. In response to the country’s transition to a super-aged society and increasing per capita pharmaceutical spending, the South Korean government has adopted a selective listing policy to prioritize cost-effectiveness and evidence-based therapies. This trial was commissioned by the Ministry of Food and Drug Safety (K-MFDS) as part of a national initiative to re-evaluate the clinical utility of legacy drugs such as oxiracetam in high-risk populations, including patients with PSCI.^3^

Oxiracetam, a pyrrolidone derivative, is thought to enhance cognition by stimulating phospholipid biosynthesis, stabilizing neuronal membranes, and promoting cholinergic transmission^4^ – mechanisms potentially relevant in PSCI, where cholinergic deficits and ischemic damage are prevalent.^5^ The dose tested (800 mg twice daily) corresponds to the standard approved regimen in South Korea. Although potential benefits have been reported in Alzheimer’s disease and multi-infarct dementia,^6–8^ clinical evidence in PSCI remains sparse and methodologically inconsistent.

We hypothesized that concurrent physical activity could augment oxiracetam’s effects via shared mechanisms such as hippocampal plasticity and improved cerebral perfusion.^9,10^ The FINGER trial and other multidomain interventions suggest that combining pharmacologic and lifestyle strategies may offer additive benefits.^11^ Accordingly, this trial included a structured physical activity component and monitored participants using wrist-worn actigraphy. To further elucidate related mechanisms, network-level cognitive function was assessed using resting-state functional MRI (rs-fMRI), focusing on measures of functional segregation and integration.^12^

To increase the likelihood of detecting cognitive change, we targeted high-risk individuals for PSCI– advanced age, recurrent stroke, diabetes, atrial fibrillation, and moderate-to-severe white matter hyperintensities (WMH). Prior trials such as PRoFESS and SPS3, which investigated antiplatelet regimens for secondary stroke prevention, also examined cognitive outcomes but failed to show significant treatment effects.^13,14^ Critical reviews of these studies have noted that the lack of observable cognitive decline in both arms may be attributed to the inclusion of predominantly low-risk participants. Based on these insights, we enrolled individuals with established PSCI risk factors to increase the probability of cognitive deterioration during follow-up and thereby better assess potential treatment effects.

This multicenter trial aimed to evaluate whether oxiracetam prevents cognitive decline in high-risk post-stroke patients and whether sustained physical activity influences treatment response. Exploratory neuroimaging analyses were also conducted to investigate underlying mechanisms.

## Patients and methods

### Study design, participants, and interventions

This multicenter, double-blind, placebo-controlled trial was conducted at 30 university or general hospitals across South Korea. Eligible participants were aged ⩾50 years, at least 3 months post-stroke, and reported subjective cognitive decline, with one or more additional high-risk profiles for PSCI (Supplemental Table S1).^15^ These high-risk profiles– such as subjective cognitive decline, older age, recurrent stroke, and small vessel disease– were chosen to enrich for individuals likely to decline over 36 weeks.^13^ Subjective cognitive decline was assessed using a stepwise protocol. Participants were asked: “Do you think you have had problems with your memory or other thinking abilities since your stroke?” Patients responding “yes” were further screened using the Subjective Cognitive Decline Questionnaire (SCD-Q),^16^ and the Clinical Dementia Rating (CDR). Those scoring ⩾7 on the SCD-Q or ⩾0.5 on the CDR memory domain were considered eligible. These criteria are detailed in Supplemental Table S1.

Key exclusion criteria included prior oxiracetam or cognitive enhancer use within 1 month, baseline dementia, major neurological or psychiatric illness, severe stroke disability (modified Rankin Scale (mRS) ⩾3 at screening), or unstable medical conditions (Supplemental Table S1). The mRS ranges from 0 (no symptoms) to 5 (severe disability); 6 indicates death.^17^

**Figure 1.**
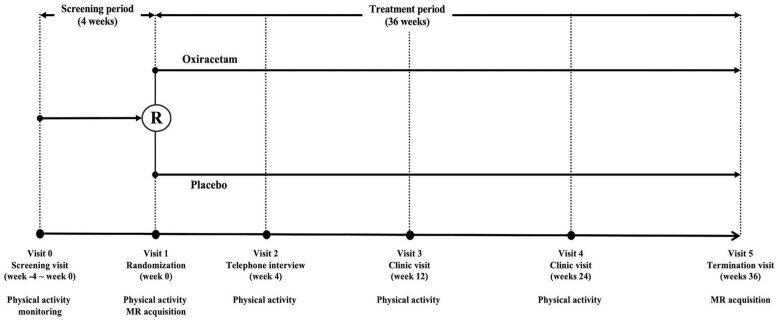
Overview of scheduled visits, assessments, and follow-up timeline.

Participants were randomized 1:1 to receive oxiracetam 800 mg or placebo twice daily for 36 weeks using a stratified block randomization (Figure 1). Stratification was based on site and education (<12 vs ⩾12 years) due to potential confounding.^18,19^ Allocation was concealed via a web-based system. Recruitment spanned from February 2018 to September 2020.

The design adhered to principles of causal inference framework, with randomization employed to minimize confounding. Physical activity was not randomized but uniformly promoted and monitored using wrist actigraphy. Full design details are provided elsewhere.^20^

Sample size calculations targeted 80% power at a 5% significance level. For MMSE, 120 participants per group were needed to detect a 1.1-point difference (SD = 3.02), and for CDR-SB, 227 per group to detect a 0.19-point difference (SD = 0.71). Accounting for 10% attrition, 506 participants were planned.^20^ Analyses were performed on the modified intention-to-treat (ITT) population – defined as those who received ⩾1 dose of study drug and had both baseline and 36-week MMSE and CDR-SB scores. Safety was evaluated in all participants who received at least one dose of the study drug.

The study was approved by institutional review boards and conducted in accordance with the Declaration of Helsinki. All participants gave written informed consent.

### Physical activity

At screening (Visit 0), participants were equipped with wrist-worn actigraphy devices (FITMETER, 32 Hz triaxial accelerometer) and individualized exercise plans based on American College of Sports Medicine and American Heart Association guidelines.^21^

Exercise programs included aerobic, strength, flexibility, and balance components, tailored by baseline mRS. Those with mRS 0–1 performed unassisted standing exercises; those with mRS 2 performed supported modifications. This stratification promoted both safety and comparability (see Supplemental Materials).

Participants received instructional materials and were assessed at weeks 0, 4, 12, 24, and 36. The target was to maintain moderate-to-vigorous physical activity (MVPA) ⩾ 20 min/day (⩾3 METs).^21^ Participants were categorized as active or inactive based on this threshold,^21^ and adherence was supported by regular feedback from study coordinators.

### Endpoints assessments

The co-primary endpoints were changes in Mini-Mental State Examination (MMSE) and Clinical Dementia Rating–Sum of Boxes (CDR-SB) from baseline to week 36. Secondary endpoints included changes in: Korean-Vascular Cognitive Impairment Harmonization Standards–Neuropsychology Protocol (K-VCIHS-NP), Korean Instrumental Activities of Daily Living (K-IADL), Neuropsychiatric Inventory (NPI), Short Geriatric Depression Scale (SGDS), EuroQoL, and Patient Global Assessment (PGA).

K-VCIHS-NP scores were standardized using Korean norms.^22^ A composite cognitive z-score was calculated across four domains: executive, language, visuospatial, and memory (Supplemental Methods). Higher MMSE and z-scores indicate better cognition; lower CDR-SB, NPI, SGDS, K-IADL, EuroQoL, and PGA scores reflect improvement.

To estimate minimum clinically important differences (MCIDs), anchor-based analyses were performed using week 36 PGA responses.^20,23^ In PGA, participants responded to the question: “How does your condition compare to before you started treatment with the study drug?” using a 7-point scale (1 = significantly improved to 7 = significantly worse). For oxiracetam, MCIDs were 0.47 (MMSE) and −0.25 (CDR-SB) for PGA ratings of 2–3 (moderate/mild improvement) as anchors, and 0.53 and −0.45, respectively, for ratings of 1–2 (significant/moderate improvement) as anchors.

MRI included T1, rs-fMRI, and DTI with harmonized protocols (Supplemental Table S2). Functional network parameters (e.g. global efficiency, clustering coefficient) were computed and analyzed as secondary outcomes.^24^ Network parameters were normalized to null distributions and assessed using paired *t*-tests or Wilcoxon signed-rank tests.

### Statistical analysis

Baseline characteristics were summarized as means ± SD or medians (IQR) for continuous variables, and as counts (percentages) for categorical variables. Group comparisons were performed using Student’s *t*-test for normally distributed continuous variables, the Mann–Whitney *U* test for skewed data, and chi-square or Fisher’s exact tests for categorical variables. Normality was assessed using the Shapiro–Wilk test and visual inspection of histograms.

The co-primary endpoints were analyzed using Student’s *t*-test or the Mann–Whitney *U* test. Two-way ANOVA was prespecified to adjust for MVPA imbalance. Secondary endpoints were evaluated using paired and Student’s *t*-tests or Wilcoxon signed-rank tests.

Multivariable linear mixed models were used to adjust for baseline covariates if imbalances remained despite randomization (*p* < 0.10), with center as a random effect and education as a fixed effect. Significant treatment-by-covariate interactions were retained in the final models.

As sensitivity analysis, effect modification by physical activity was assessed using MVPA tertiles and two-way ANOVA. MVPA was also modeled continuously in ANCOVA. A sequential conditional mean model (SCMM) accounted for repeated MVPA measurements. Regression models tested interaction terms between treatment and physical activity (active/inactive, or MVPA tertile; see Supplemental Methods).

Participants with missing MMSE or CDR-SB scores at week 36 were excluded from the modified ITT analysis. No imputation was performed for cognitive outcomes. For missing physical activity data (~13%, mainly due to device malfunction), median imputation was performed within strata defined by treatment group and education level.

All statistical tests were two-sided, with a significance threshold of 0.05 for primary and secondary endpoints. A p-value < 0.10 was used to guide the inclusion of covariates in multivariable models and interaction terms in exploratory analyses.^25,26^ All analyses were conducted using SAS^®^ version 9.4 (SAS Institute, Cary, NC, USA).

## Results

### Participants

**Figure 2.**
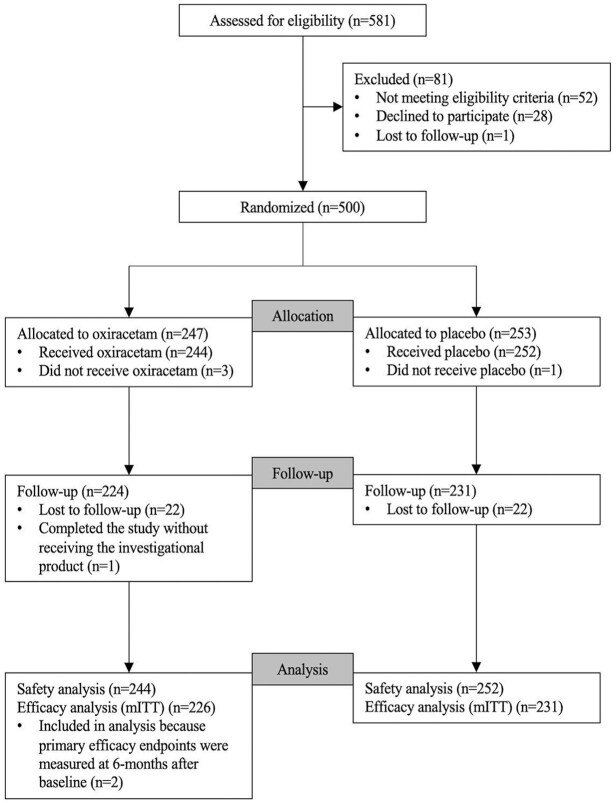
CONSORT diagram showing participant enrollment, randomization, and analysis populations. The follow-up population includes participants who received at least one dose of study drug and completed both baseline and 36-week cognitive assessments.

A total of 500 patients were randomized to oxiracetam (*n* = 247) or placebo (*n* = 253). The modified ITT population included 457 participants, excluding one who did not initiate treatment and 42 lacking co-primary endpoint data (Figure 2). The median time from stroke to randomization was 2.61 years, slightly shorter in the oxiracetam group (2.2 vs 2.6 years).

Mean age was 68.8 ± 8.6 years (oxiracetam) and 69.0 ± 8.9 years (placebo); women comprised 24.8% and 22.9%, respectively. The trial population (*n* = 457) showed high PSCI risk: 51% were aged ⩾70, 16% had recurrent stroke, 44% had diabetes, and 24% had moderate-to-severe WMH, with no significant differences between the treatment groups.

Medication adherence was high and similar across groups (oxiracetam: 91%, placebo: 92%, *p* = 0.46). Baseline imaging showed more cortical involvement in the oxiracetam group (43.2% vs 35.6%), while prior cerebral hemorrhage was more frequent in the placebo group (3.9% vs 0.9%). Depressive symptoms (SGDS ⩾ 8) were reported in 29.6% of oxiracetam and 24.7% of placebo participants (*p* = 0.23), with no significant group difference at baseline.

### Feasibility of physical activity monitoring

Wrist actigraphy successfully recorded activity in 87% of participants. MVPA adherence was similar (oxiracetam: 65.5%, placebo: 65.8%, *p* = 0.94) with no significant difference in tolerability. Mean daily MVPA duration was also comparable (oxiracetam: 23.2 ± 23.0 min; placebo: 24.1 ± 28.7 min; *p* = 0.75; Table 1, Supplemental Table S3).

**Table 1.** Baseline characteristics of participants (modified ITT population, *N* = 457).

Characteristic	Oxiracetam (*n* = 226)	Placebo (*n* = 231)	Total (*n* = 457)
Age, years	68.8 ± 8.6	69.0 ± 8.9	68.9 ± 8.7
⩾70 years old	114 (50.4%)	120 (51.9%)	234 (51.2%)
Female	56 (24.8%)	53 (22.9%)	109 (23.9%)
Education, years (median, IQR)	10.5 (6.0, 12.0)	12.0 (6.0, 12.0)	11.0 (6.0, 12.0)
Elementary school	67 (29.7%)	62 (26.8%)	129 (28.2%)
Middle school	43 (19.0%)	49 (21.1%)	92 (20.1%)
High school	74 (32.7%)	74 (32.0%)	148 (32.4%)
University or above	42 (18.6%)	46 (19.9%)	88 (19.3%)
Interval from stroke onset to randomization, months (median, IQR)	28.3 (12.4, 73.8)	34.3 (12.9, 70.8)	31.7 (12.7, 71.7)
Hypertension	182 (80.5%)	186 (80.5%)	368 (80.5%)
Diabetes mellitus	101 (44.7%)	100 (43.3%)	201 (44.0%)
Dyslipidemia	156 (69.0%)	154 (66.7%)	310 (67.8%)
Atrial fibrillation/flutter	45 (19.9%)	38 (16.5%)	83 (18.2%)
Ischemic heart disease	27 (11.9%)	21 (9.1%)	48 (10.5%)
Heart failure	4 (1.8%)	6 (2.6%)	10 (2.2%)
Current smoking	40 (16.2%)	52 (20.6%)	92 (20.1%)
Body mass index (kg/m^2^)	25.0±3.3	24.5±3.0	24.7±3.1
Systolic blood pressure	128.3±14.5	130.2±13.4	129.3±14.0
Diastolic blood pressure	74.5±9.9	74.6±9.4	74.5±9.6
Heart rates	77.8±11.4	75.8±11.9	76.8±11.7
History of recurrent stroke	35 (15.5%)	36 (15.6%)	71 (15.5%)
Index stroke TOAST			
LAA	82 (36.3%)	79 (34.2%)	161 (35.2%)
CE	38 (16.8%)	38 (16.5%)	76 (16.6%)
SVO	70 (31.0%)	73 (31.6%)	143 (31.3%)
OD	15 (6.6%)	14 (6.1%)	29 (6.3%)
UD	21 (9.3%)	23 (10.0%)	44 (9.6%)
Index stroke NIHSS (median, IQR)	2.0 (1.0, 4.0)	3.0 (1.0, 5.0)	2.0 (1.0, 4.0)
Baseline NIHSS (median, IQR)	0 (0, 1.0)	0 (0, 1.0)	0 (0, 1.0)
Baseline mRS, 0/1/2	105 (46.5%)/97 (42.9%)/ 24 (10.6%)	115 (49.8%)/86 (37.2%)/ 30 (13.0%)	220 (48.1%)/183 (40.0%)/54 (11.8%)
Index stroke imaging: acute			
Cortical involvement	96 (43.2%)	79 (35.6%)	175 (38.3%)
Left-hemispheric involvement	125 (50.6%)	139 (54.9%)	264 (57.8%)
Multiple lesions	108 (48.6%)	106 (47.7%)	214 (46.8%)
Index stroke imaging: chronic			
Periventricular WMH, Fazekas 0/1/2/3	0 (0.0%)/114 (54.3%)/ 58 (27.6%)/38 (18.1%)	0 (0.0%)/118 (54.1%)/56 (25.7%)/44 (20.2%)	0 (0.0%)/232 (50.8%)/ 114 (24.9%)/82 (17.9%)
Subcortical WMH, Fazekas 0/1/2/3	19 (9.0%)/ 139 (66.2%)/ 43 (20.5%)/ 9 (4.3%)	22 (10.1%)/123 (56.4%)/ 61 (28.0%)/ 12 (5.5%)	41 (9.0%)/262 (57.3%)/104 (22.8%) / 21 (4.6%)
Number of cerebral microbleeds	4.12±7.78	5.14±11.39	4.63±9.78
Number of lacunes	2.23±3.38	2.43±3.19	2.33±3.29
Cerebral hemorrhage	2 (0.9%)	9 (3.9%)	11 (2.4%)
Chronic territorial infarction	15 (6.6%)	15 (6.5%)	30 (6.6%)
Medial temporal atrophy, Scheltens 0/1/2/3/4	90 (40.0%)/ 57 (25.3%)/ 68 (30.2%)/10 (4.4%)/ 0 (0%)	86 (37.7%) / 63 (27.6%)/ 65 (28.5%)/ 12 (5.3%)/ 2 (0.9%)	176 (38.5%) / 120 (26.3%)/ 133 (29.1%)/22 (4.8%)/ 2 (0.4%)
Global cortical atrophy 2 or more	46 (22.3%)	35 (17.4%)	81 (17.7%)
SCD-Q scores	11.69±4.35	11.11±3.85	11.39±4.11
Mini-Mental State Examination	26.58±2.57	26.64±2.60	26.61±2.58
CDR-sum of boxes			
0	32 (14.2%)	34 (14.7%)	66 (14.4%)
0.5	185 (81.9%)	189 (81.8%)	374 (81.8%)
1.0	9 (4.0%)	8 (3.5%)	17 (3.7%)
SGDS ⩾ 8	67 (29.6%)	57 (24.7%)	124 (27.1%)
Neuroimaging (normalized)			
Global efficiency	0.85±0.03	0.85±0.03	0.85±0.03
Characteristic path length	1.26±0.07	1.27±0.07	1.26±0.07
Clustering coefficient	1.73±0.28	1.74±0.27	1.74±0.28
Modularity	3.35±0.41	3.36±0.39	3.35±0.40
Physical activity levels			
Baseline MVPA duration, minutes	23.2±23.0	24.1±28.7	23.7±26.1
Baseline MVPA duration ⩾ 20 min	148 (65.5%)	152 (65.8%)	300 (65.6%)

ITT: intention-to-treat; IQR: interquartile range; TOAST: Trial of ORG 10172 in Acute Stroke Treatment; LAA: large artery atherosclerosis; CE: cardioembolism; SVO: small vessel occlusion; OD: other determined; UD: undetermined; NIHSS: National Institutes of Health Stroke Scale; mRS: modified Rankin scale; WMH: white matter hyperintensities; SCD-Q: subjective cognitive decline questionnaire; SGDS: short version of the geriatric depression scale; MVPA: moderate-to-vigorous physical activity.

Numbers denote mean ± standard deviations or median (IQR) for continuous variables or frequencies (proportions) for categorical variables.

### Efficacy outcomes

**Figure 3.**
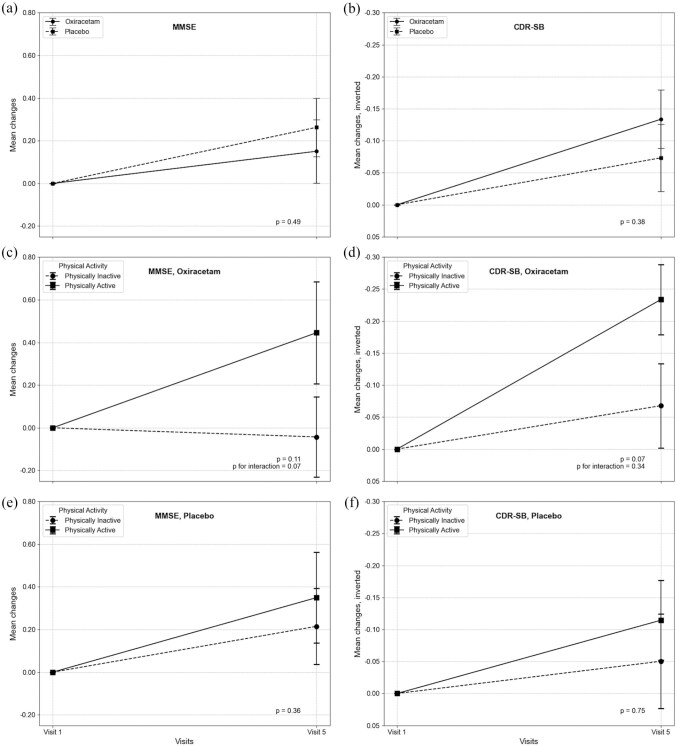
Changes in MMSE and CDR-SB scores from baseline (Visit 1) to week 36 (Visit 5) by treatment allocation and baseline physical activity status (*N* = 457) Error bars represent standard errors (SE) of the mean changes. (a and b) Mean changes in MMSE and CDR-SB scores for the oxiracetam and placebo groups, respectively. (c and d) Mean changes in MMSE and CDR-SB scores among participants allocated to oxiracetam, stratified by baseline physical activity (Physically Active vs Physically Inactive). (e and f) Corresponding mean changes in the placebo group. For clarity, the direction of the CDR-SB was inverted so that upward trends represent cognitive improvement, as with the MMSE. As physical activity at baseline was balanced across randomized groups, the mean changes in MMSE and CDR-SB scores (follow-up [V5] − baseline scores [V1]) between treatment groups were compared using Student’s *t* test. In addition, the significance of the interaction effect between treatment group and baseline physical activity was tested using a 5% significance level through multiple linear regression analysis with sequential conditional mean model (SCMM). Physically Active was defined as engaging in ⩾20 min/day of MVPA at baseline; Physically Inactive was defined as <20 min/day of MVPA. Abbreviations: MMSE, mini-mental state examination; CDR-SB, clinical dementia rating-sum of boxes; MVPA, moderate-to-vigorous physical activity.

As baseline MVPA did not differ significantly (*p* = 0.94), co-primary endpoints were compared using Student’s t-tests. No significant between-group differences were observed in changes from baseline to week 36 in MMSE (+0.13 ± 2.27 vs +0.27 ± 2.09; *p* = 0.49) or CDR-SB (–0.14 ± 0.70 vs −0.08 ± 0.80; *p* = 0.38; Tables 1 and 2, Figure 3). These changes did not exceed the MCID thresholds (0.47–0.53 for MMSE; –0.25 to –0.45 for CDR-SB). MMSE improvement ⩾2 points was observed in 25% of the oxiracetam group and 21% of the placebo group (Supplemental Figure S1).

**Table 2.** Mean changes in primary and secondary outcomes from baseline to the end of the study (*N* = 457).

Endpoints	Oxiracetam (*N* = 226)	Placebo (*N* = 231)	*p*-Value^*^	*LMM* *p*-Value^†^	*SCMM* *p*-Value^‡^	*Interaction* *p*-Value^§^
Primary endpoints						
MMSE^¶^	0.13 ± 2.27	0.27 ± 2.09	0.49	0.50	0.43	0.07
CDR-SB^¶^	−0.14 ± 0.70	−0.08 ± 0.80	0.38	0.40	0.51	0.34
*Secondary endpoints*						
SVLT-E immediate recall *z*-score	0.28 ± 0.85	0.28 ± 0.80	0.69	0.99	0.95	0.09
SVLT-E delayed recall *z*-score	0.28 ± 0.87	0.18 ± 0.83	0.21	0.13	0.47	0.46
SVLT-E recognition *z*-score	0.18 ± 1.17	0.24 ± 1.17	0.59	0.32	0.92	0.24
Semantic fluency *z-*score	0.01 ± 0.78	0.03 ± 0.75	0.85	0.99	0.46	0.98
Phonemic fluency *z*-score	0.15 ± 0.69	0.17 ± 0.71	0.78	0.95	0.63	0.55
Digit symbol coding *z*-score	0.08 ± 0.60	0.06 ± 0.50	0.82	0.65	0.57	0.11
TMT-E part A *z*-score	0.004 ± 1.13	0.16 ± 1.11	0.14	0.37	0.13	0.11
TMT-E part B *z*-score	0.12 ± 1.59	−0.03 ± 1.24	0.25	0.30	0.30	0.46
Short BNT *z*-score	0.23 ± 1.10	0.14 ± 0.74	0.34	0.43	0.07	0.60
RCFT *z*-score	0.15 ± 1.22	0.004 ± 1.13	0.19	0.10	0.09	0.052
NPI-Q	−0.49 ± 3.31	−0.46 ± 2.97	0.95	0.99	0.71	0.64
SGDS	−0.88 ± 3.46	−1.00 ± 2.88	0.69	0.50	0.51	0.20
IADL	−0.01 ± 0.20	−0.03 ± 0.18	0.27	0.27	0.43	0.84
Memory domain z-score	0.25 ± 0.71	0.23 ± 0.66	0.85	0.97	0.73	0.63
Frontal domain z-score	0.07 ± 0.58	0.08 ± 0.46	0.92	0.79	0.86	0.19
Global cognitive function z-score	0.18 ± 0.51	0.11 ± 0.39	0.16	0.14	0.04	0.22
EuroQoL	−0.24 ± 1.13	−0.22 ± 1.41	0.85	0.99	0.90	0.17
PGA at Visit 5	3.60 ± 0.82	3.53 ± 0.85	0.37	0.53		
Significantly improved	5 (2.2%)	5 (2.2%)	0.86^\\^			
Moderately improved	14 (6.2%)	22 (9.5%)				
Mildly improved	64 (28.3%)	64 (27.7%)				
Almost the same	128 (56.6%)	127 (55.0%)				
Mildly worse	13. (5.8%)	11 (4.8%)				
Moderately worse	2 (0.9%)	2 (0.9%)				
Significantly worse	0 (0%)	0 (0%)				
Global efficiency	−0.004±0.038	−0.002±0.039	0.55	0.94	0.52	0.70
Characteristic path length	0.005±0.089	0.002±0.085	0.73	0.86	0.36	0.81
Clustering coefficient	0.036±0.330	0.015±0.344	0.52	0.04	0.20	0.96
Modularity	0.039±0.484	0.016±0.524	0.63	0.96	0.76	0.73

LMM: linear mixed model; SCMM: sequential condition mean model; MMSE: mini-mental state examination; CDR-SB: clinical dementia rating-sum of boxes; SVLT-E: Seoul verbal learning test-elderly version; TMT-E: trail-making test-elderly version; BNT: Boston naming test; RCFT: Rey complex figure test; NPI-Q: neuropsychiatric inventory questionnaire; SGDS: short version of the geriatric depression scale; IADL: instrumental activities of daily living; EuroQoL: European quality of life-5 dimensions; PGA: patient’s global assessment.

Numbers denote mean ± standard deviations or median (IQR) for continuous variables or frequencies (proportions) for categorical variables.

^*^ *p*-Value by Student’s *t-*test, unless otherwise specified.

^†^ *p*-Value by linear mixed model treating center effect as random using independence variance-covariance structure.

^‡^ *p*-Value for group difference of efficacy measurement after adjusting baseline efficacy measurement and time-dependent confounders of follow-up #2 and #3 MVPA time effect using multiple linear regression model (SCMM) without interaction (analyzed only when the interaction *P*-value of SCMM with interaction model is ⩾0.05). Dependent variable: visit 5 efficacy measurements, independent variables: group, baseline efficacy measurement, follow-up #2 MVPA time) and exercise follow-up #3 MVPA time.

^§^“Treatment group” by “baseline MVPA degree” interaction *P*-value for multiple linear regression model (SCMM) with interaction [dependent variable: visit 5 efficacy measurements; independent variables: treatment group, baseline efficacy measurement, baseline MVPA time, follow-up #2 MVPA time, follow-up #3 MVPA time, and interaction between treatment group and baseline MVPA time].

^¶^Change = visit 5 (week 36) – baseline value.

^\\^ *p*-Value by Fisher’s exact test.

Secondary endpoints were comparable between groups, though the study was not powered to detect differences in these measures (Table 2). Within-group improvements were noted in global cognitive z-scores (Supplemental Table S4): oxiracetam from −0.49 ± 0.74 to −0.32 ± 0.79, and placebo from −0.46 ± 0.79 to −0.35 ± 0.82 (both *p* < 0.01). A significant within-group reduction in CDR-SB was observed in the oxiracetam group but not in the placebo group.

In multivariable generalized linear mixed model analyses, adjusting for site, education, and baseline imbalances (*p* < 0.10), no significant treatment effects were detected for primary or secondary endpoints. However, among brain network metrics, the normalized clustering coefficient was higher in the oxiracetam group compared to placebo (adjusted mean difference in clustering coefficient [β] = 0.09, SE = 0.04, *p* = 0.04; Supplemental Table S5).

In exploratory sensitivity analysis, participants in the highest MVPA tertile receiving oxiracetam showed greater CDR-SB improvement (–0.28 ± 0.55 vs −0.10 ± 0.50, *p* < 0.05; Supplemental Figure S2). However, the treatment-by-MVPA interaction was not significant (*p* = 0.76).

### Safety outcomes

Safety was assessed in all 496 participants who received at least one dose. Adverse events occurred in 41.0% of the oxiracetam group (100/244) and 34.9% of the placebo group (88/252; *p* = 0.16; Table 3). While not statistically significant, this represents a numerically higher incidence in the oxiracetam group. No clinically relevant safety signals attributable to oxiracetam were identified.

**Table 3.** Adverse events (safety population, *N* = 496).

Adverse events	Oxiracetam (*n* = 244)	Placebo (*n* = 252)	*p* Value
Any AE	100 (41.0%)	88 (34.9%)	0.16
Any serious AE	21 (8.6%)	16 (6.3%)	0.34
Any serious AE causing treatment discontinuation	3 (1.2%)	1 (0.4%)	0.46
Serious AE by SOC term			
Cardiac	3 (1.2%)	2 (0.8%)	0.43
Gastrointestinal	1 (0.4%)	3 (1.2%)	0.99
Hepatobiliary	1 (0.4%)	0 (0%)	n/a
Infections	1 (0.4%)	1 (0.4%)	n/a
Injury, poisoning and procedural complications	1 (0.4%)	4 (1.6%)	0.74
Musculoskeletal and connective tissue disorders	4 (1.6%)	2 (0.8%)	0.16
Neoplasm	4 (1.6%)	3 (1.2%)	0.15
Nervous system	4 (1.6%)	1 (0.4%)	0.74
Renal and urinary	0 (0%)	1 (0.4%)	n/a
Reproductive and breast	1 (0.4%)	0 (0%)	n/a
Skin and subcutaneous tissue	0 (0%)	1 (0.4%)	n/a
Vascular	1 (0.4%)	0 (0%)	n/a

AE: adverse events; SOC: system organ class; MedDRA: medical dictionary for regulatory activities.

A participant can have more than one AE within the same SOC. In this case, the event rate is considered and calculated as one SOC, while the number of events is calculated for each AE. MedDRA dictionary Version 25.0 was used for coding.

## Discussion

This multicenter randomized controlled trial evaluated whether oxiracetam prevents PSCI in high-risk patients with subjective cognitive decline, and whether sustained physical activity modifies its effect. Oxiracetam did not show superior efficacy over placebo in preventing PSCI. Across 457 participants with previous stroke and elevated PSCI risk, cognitive function was generally preserved or improved over the 36-week follow-up. Although participants in the highest physical activity tertile showed greater cognitive improvements, no statistically significant interaction with oxiracetam was found.

The trial design incorporated causal inference principles, with randomization aimed to reduce confounding. Physical activity was standardized and closely monitored to support exploratory analyses. However, since it was not randomized, any related findings should be considered hypothesis-generating, not causal. Future trials using factorial designs are needed to examine its interactive effects more definitively.

Our findings did not replicate earlier reports of oxiracetam efficacy in PSCI.^6,7^ We hypothesized additive or synergistic effects with physical activity, but found none. While physical activity is generally associated with cognitive benefit, emerging data suggest its effects may vary by intensity and duration, with some studies reporting neutral or adverse outcomes.^27,28^ Including relatively active participants may have led to selection bias, favoring healthier individuals and attenuating observable treatment effects. Prior research links preserved mobility with better cognition,^29^ suggesting physical activity alone may have contributed to cognitive stability across both groups.

Compared to earlier trials, our participants demonstrated minimal cognitive decline. Rozzini et al. reported a 1.3-point MMSE gain with oxiracetam versus 0.2 with placebo over 2 months.^6^ In contrast, we observed only a 0.13-point gain in the oxiracetam group and 0.27-point gain in the placebo group over 9 months. The higher baseline MMSE in our cohort (mean 26.6 vs 22.1 in Rozzini et al.) likely limited measurable improvement.^6^ Our sample size was based on Winblad et al., who found a 0.19-point CDR-SB difference between galantamine and placebo in MCI.^30^ While PSCI differs from MCI, we deemed them comparable in risk and cognitive status. Nonetheless, both groups in our trial improved (–0.14 vs −0.08), yielding only a 0.06-point difference– far smaller than anticipated.

Notably, 21% of placebo participants improved ⩾2 MMSE points, highlighting the possible role of non-pharmacologic factors, such as sustained physical activity. Nearly 60% met target MVPA levels, and activity adherence was supported through actigraphy feedback and educational resources. Although the trial was not powered to assess physical activity effects, greater CDR-SB improvement in the most active oxiracetam subgroup (–0.28 vs −0.10, *p* = 0.0459) was observed. However, interaction testing was not significant (*p* = 0.76), and these exploratory findings warrant caution. Still, the feasibility and acceptability of long-term activity in stroke survivors were demonstrated, offering direction for future intervention trials.

Among rs-fMRI metrics, only the clustering coefficient – reflecting local segregation – differed between groups, favoring oxiracetam. This metric captures regional processing efficiency and has been linked to cognition.^24,31^ Despite standardized protocols and quality control, scan variability and participant factors may have limited detection of other network differences.

To minimize practice effects, MMSE and CDR-SB were assessed only at baseline and week 36. Prior research suggests that learning effects wane after the initial 3 months.^32^ The 9-month interval likely reduced this bias.

Several limitations should be noted. First, actigraphy data were missing in 13% due to device failure. Although largely technical, missingness may not be random – patients with cognitive decline may have had lower adherence, potentially biasing results.

Second, women comprised ~20% of the cohort, reflecting persistent sex imbalance in stroke trials.^33^ Reasons may include higher comorbidity, lower participation rates, or recruitment bias. While physical activity was standardized, its demands may have disproportionately deterred women. Future studies should adopt sex-sensitive designs to improve representativeness and equity in trial participation.

Third, MMSE has limited sensitivity for post-stroke cognitive deficits, particularly executive dysfunction, attention deficits, and delayed recall.^34^ Ceiling effects in our relatively high-functioning cohort likely blunted its responsiveness. While CDR-SB and domain-specific scores showed improvement, MMSE changes remained modest. Future studies should consider more sensitive, stroke-specific instruments.

Our eligibility criteria targeted individuals with subjective cognitive decline and additional risk factors for PSCI, including advanced age, recurrent stroke, diabetes, atrial fibrillation, and moderate-to-severe WMH. While this approach enriched the sample for those at higher risk of cognitive decline, it may have introduced selection bias toward individuals with more advanced neurodegenerative burden, potentially limiting the observable treatment effect. Furthermore, requiring self-reported cognitive concerns may have excluded individuals with impaired awareness or lower concern about their symptoms. Conversely, this criterion may have favored the inclusion of individuals with relatively preserved cognition and heightened health awareness – so-called “worried well” – who tend to have a more favorable prognosis. As a result, our cohort may not fully represent the broader spectrum of post-stroke cognitive trajectories, and caution is warranted in generalizing these findings.

In conclusion, this randomized trial did not support the efficacy of oxiracetam in preventing cognitive decline among high-risk post-stroke patients. In January 2023, the K-MFDS issued a drug safety communication recommending the suspension of oxiracetam prescriptions. These findings underscore a broader challenge faced by aging societies worldwide: the imperative to ensure that pharmaceutical use is both evidence-based and economically sustainable. Our results may inform similar drug reassessment initiatives in other countries, particularly in guiding the reevaluation of legacy medications and the development of cost-conscious, value-driven health policy frameworks.

## Supplementary Material

sj-docx-1-eso_23969873251350141

## Data Availability

Data from this study is accessible upon reasonable request to the corresponding author.

## Trial registration

URL: https://cris.nih.go.kr/cris/index/index.do; Unique Identifier: KCT0005137.

## ORCID iDs

Jae-Sung Lim   https://orcid.org/0000-0001-6157-2908

Jong-Ho Park   https://orcid.org/0000-0002-2681-1878

Sung Hyuk Heo   https://orcid.org/0000-0002-9215-5119

Hahn Young Kim   https://orcid.org/0000-0001-9591-4005

Keun-Hwa Jung   https://orcid.org/0000-0003-1433-8005

Chulho Kim   https://orcid.org/0000-0001-8762-8340

Jong-Moo Park   https://orcid.org/0000-0002-4199-3024

Jun Hong Lee   https://orcid.org/0000-0002-5023-6009

Jong-Won Chung   https://orcid.org/0000-0002-9200-8899

Hee-Joon Bae   https://orcid.org/0000-0003-0051-1997
