# Supplementary material for: Oxiracetam and physical activity in preventing cognitive decline after stroke: A multicenter, randomized controlled trial
Source: Eur Stroke J. 2026 Jan 1;11(1):23969873251350141. doi: 10.1093/esj/23969873251350141 (PMC12866270; doi:10.1093/esj/23969873251350141)
Supplement: sj-docx-1-eso_23969873251350141 [file sj-docx-1-eso_23969873251350141.docx]

**Supplemental materials**

**Supplemental methods**

**Korean-Vascular Cognitive Impairment Harmonization Standards-Neuropsychology Protocol (K-VCIHS-NP)**

The K-VCIHS-NP is comprised of eight cognitive evaluations and four cognitive domains. The following exams are administered for each cognitive domain: 1) Semantic fluency (animal),^1^ phonemic fluency (Korean-Controlled Oral Word Association Test),^1^ Digit Symbol Coding,^2^ and Korean-Trail Making Test-Elderly's version-Parts A and B^3^ for the frontal-executive domain; 2) Korean-Boston Naming Test for the language domain;^4^ 3) Rey Complex Figure Test: Copy.^5^ The frontal domain score was determined by averaging the z-scores of the five subtest measures. On the Seoul Verbal Learning Test, the memory score was calculated as the mean of the three z-scores. The global cognitive score was derived by aggregating the z-scores from the frontal-executive, language, visuospatial, and memory domains.

### Physical Activity Stratification Based on mRS

At baseline (Visit 0), functional status was assessed using the modified Rankin Scale (mRS), a validated clinical outcome measure of post-stroke disability ranging from 0 (no symptoms) to 5 (severe disability requiring constant care). Exercise prescriptions were individualized based on mRS scores and in accordance with American College of Sports Medicine and the American Heart Association and included four components: aerobic activity, strength training, flexibility exercises, and balance training.

For all participants, the general recommendations were as follows:

- **Aerobic activity:** 3–7 sessions per week, starting from 20 minutes per session and increasing up to 60 minutes as tolerated. The target intensity was light to moderate, defined as slightly breathless or sweating, with a target heart rate of approximately 90–120 beats per minute.
- **Strength training:** 2–3 sessions per week, consisting of 1–3 sets of 10–15 repetitions.
- **Flexibility training:** 2–3 sessions per week, holding each stretch for 10–30 seconds.
- **Balance training:** 2–3 sessions per week, incorporating coordination and stability exercises.

Physical activity recommendations were then tailored according to each participant’s functional level:

#### Participants with mRS 0–1 (No or minimal disability):

- **Aerobic activities:** brisk walking, stair climbing, water-based exercise, aerobics, step exercises, badminton, dance, traditional Korean dance, hiking, and rhythmic clapping with marching in place.
- **Strength training:** sit-to-stand repetitions, squats.
- **Flexibility exercises:** lower-limb stretching.
- **Balance training:** tandem walking, single-leg standing, and dynamic balance strengthening.

#### Participants with mRS 2 (Slight disability):

- **Aerobic activities:** casual walking, leisure walking, light housework, choosing stairs over elevators, increasing outings, walking with a pet, walking to do light shopping, walking as the preferred mode of transportation, standing and moving every 20 minutes during prolonged sitting, and participating in community activities.
- **Strength training:** thigh-strengthening exercises.
- **Flexibility exercises:** seated or standing flexibility routines.
- **Balance training:** single-leg standing and tandem gait practice.

Physical activity was monitored continuously using wrist-worn accelerometers (FITMETER, 32 Hz triaxial), and all participants received identical instructional materials and ongoing support.

**Sensitivity Analyses of Physical Activity–Treatment Interactions**

1. Categorical Stratification of Baseline Physical Activity (Tertile Model)

To explore whether different levels of baseline physical activity influenced the treatment effect, baseline daily moderate-to-vigorous physical activity (MVPA) was categorized into tertiles (low, middle, high) rather than dichotomized at ≥20 minutes/day. Frequencies and percentages were summarized for each treatment group, and between-group comparisons of baseline tertile distributions were conducted using chi-square or Fisher’s exact tests. If a statistically significant difference (two-sided p < 0.10) in the distribution of MVPA tertiles between treatment groups was detected, a two-way ANOVA was performed. The model included:

- Dependent variable: change in each cognitive outcome score (MMSE or CDR-SB)
- Fixed factors: treatment group (oxiracetam vs. placebo), baseline MVPA tertile, and their interaction

If the interaction term reached statistical significance at the 5% level, stratified contrasts were conducted to compare treatment effects within each MVPA tertile. If the interaction was not significant, a reduced model excluding the interaction term was re-fitted to evaluate treatment effects adjusted for MVPA level.

2. Continuous Baseline MVPA (ANCOVA Model)

Mean daily MVPA at baseline was also analyzed as a continuous variable. Descriptive statistics (n, mean, SD, median, IQR, range) were calculated by treatment group, and group differences were tested using Student’s t-test or Mann–Whitney U test. If baseline MVPA differed significantly (p < 0.10) between groups, analysis of covariance (ANCOVA) was used to evaluate treatment group differences in cognitive change scores. The model included:

- Dependent variable: change in each cognitive outcome score (MMSE or CDR-SB)
- Independent variables: treatment group, baseline MVPA (continuous), and their interaction

If the interaction term was significant (p < 0.05), stratified contrasts were conducted across baseline MVPA levels. If not, the model was re-fitted excluding the interaction.

3. Repeated MVPA Measures (Sequential Conditional Mean Model)

To account for repeated measurements of physical activity over the trial, we conducted a sensitivity analysis using a sequential conditional mean model (SCMM). The SCMM adjusted for:

- Baseline score of each cognitive outcome (MMSE or CDR-SB)
- Treatment group
- Whether the participant met MVPA guidelines (≥20 minutes/day) during follow-up periods #2 (weeks 12–24) and #3 (weeks 24–36)
- Interaction between treatment group and MVPA status at follow-up #3

If the interaction was significant (p < 0.05), group differences were compared within each MVPA status subgroup. If not, the model was re-fitted excluding the interaction term to yield adjusted treatment effects. This approach corrects for changes in physical activity during the study period and avoids potential bias from unbalanced activity trends between groups, as recommended by Keogh et al.^6^

**Table S1. Eligibility criteria**

| **Inclusion criteria** |
| --- |
| 1. Age $\geq$ 50 years  2. 3 months after stroke  3. With subjective cognitive decline that satisfies the following conditions  Those who answered “yes” to the one-question questionnaire, “Do you think there has been a cognitive problem including memory after stroke?”  Patients with 7 or more points in subjective cognitive decline-questionnaire or 0.5 or more points in memory domain score of clinical dementia rating  4. With one or more of the following four conditions (at high risk for dementia conversion)  Aged 70 or older  History of recurrent stroke  Atrial fibrillation or diabetes mellitus  One of followings in an index-stroke image: moderate-to-severe white matter hyperintensities (Fazekas grade $\geq$ 2 both in periventricular and deep white matter hyperintensities), global cortical atrophy index $\geq$ 2, Scheltens visual grade $\geq$ 2 for medial temporal lobe atrophy  5. With a life expectancy of 12 months or more at the time of screening  6. Patients who gave written informed consent to participate in this clinical trial |
| **Exclusion criteria** |
| 1. < 6-year education  2. Modified Rankin scale score $\geq$ 3  3. Clinical dementia rating score $\geq$ 1  4. Cognitive impairment due to diseases other than stroke  5. Unable to walk or perform physical activity  6. With the significant abnormalities in the laboratory tests at the time of screening*  7. Who have taken oxiracetam, acetylcholinesterase inhibitors, memantine, choline precursors, or acetylcarnitine within one months of the clinical trial registration date  8. Who are taking drugs that may affect the cognitive function and cannot maintain stable doses for more than 30 days before the clinical trial registration date and until the end of the clinical trial  9. With severe aphasia, hearing or visual impairment that cannot be evaluated for effectiveness assessment including Mini-Mental State Examination  10.With contraindications to magnetic resonance imaging including pacemakers  11. With schizophrenia or major depression  12. With alcohol or illegal substance abuse or dependence  13. With hypersensitivity to trial drugs and pyrrolidine derivatives or other additives  14. Who participated in other clinical trials within one month of screening (observation studies without drugs are irrelevant, but clinical studies evaluating cognitive impairment are excluded)  15. Pregnant or lactating women  16. Of childbearing potential during the clinical trial period who does not consent to contraception by the method accepted in this clinical trial  17. With severe bleeding, cardiovascular and endocrine diseases that are difficult to conduct this study  18. Who have conditions that may affect the evaluation of the results of this clinical trial or who are inappropriate to participate in this clinical trial when judged by investigators. |

* aminotransferase levels exceed three times the upper limit of normal, creatinine clearance less than 15 ml/min, hemoglobin less than 8 mg/dL, platelet count less than 50,000/mm3, hemoglobin A1c > 11%, systolic blood pressure > 180 mmHg, thyroid-stimulating hormone greater than 1.5 × the upper limit of normal.

† hormonal contraception, intrauterine devices (IUDs), intrauterine systems (IUSs), vasectomy, tubal ligation, or double-barrier methods.

**Table S2. Neuroimaging acquisition parameters**

|  |  | Philips | Siemens | GE |
| --- | --- | --- | --- | --- |
| DTI | FOV | 240 mm | 240 mm | 240 mm |
|  | Flip angle | 90 | 90 | 90 |
|  | TR | 7089 | 7400 | Maximum* |
|  | TE | 82 | 89 | Minimum* |
|  | Matrix | 120*120 | 120*120 | 120*120 |
|  | Voxel size | 2*2*2 | 2*2*2 | 2*2*2 |
|  | B-factor | 1000 | 1000 | 1000 |
|  | DIR | 32 directions | 30 directions | 32 directions |
|  | Parallel factor | 2 (Sense) | 2 (Grappa) | 2 (Asset) |
|  | slices | 70 slices | 70 slices | 70 slices |
| Resting fMRI |  |  |  |  |
|  | FOV | 240 | 240 | 240 |
|  | TR | 2000 (3T) | 2000 (3T) | 2000 (3T) |
|  | TE | 30 | 30 | 30 |
|  | Matrix | 80*80 | 80*80 | 80*80 |
|  | Voxel size | 3*3*4.5 | 3*3*4.5 | 3*3*4.5 |
|  | Flip angle | 90 | 90 | 90 |
|  | Dyn scans | 180 | 180 | 180 |
|  | Slices | 31 slices | 31 slices | 31 slices |
| Volumetric 3D |  | MPRAGE or TFFE | MPRAGE | IR_FSPGR |
|  | FOV | 256 mm | 256 mm | 256 mm |
|  | TR | 8.1 | 2300 | 7.9 |
|  | TE | 4.6 | 3 (TI900) | 3 |
|  | Matrix | 256*256*176 | 256*256*176 | 256*256*176 |
|  | Voxel size | 1*1*1 | 1*1*1 | 1*1*1 |
|  | Flip angle | 8 | 9 | 12 |
|  | Parallel factor | 2 | 2 | 2 |

Abbreviations: DTI, diffusion tensor imaging; FOV, Field of View; TR, Repetition Time; TE, Echo Time; DIR, Diffusion Imaging Directions; Matrix, Acquisition Matrix; Slices, Number of Slices; Parallel factor, Parallel Imaging Acceleration Factor; Dyn scans, Dynamic Scans; MPRAGE, Magnetization Prepared Rapid Gradient Echo; TFFE, Turbo Field Echo; IR_FSPGR, Inversion Recovery–Fast Spoiled Gradient Echo; TI, Inversion Time; GE, General Electric; Asset, Array Spatial Sensitivity Encoding Technique; Sense, Sensitivity Encoding; Grappa, Generalized Autocalibrating Partial Parallel Acquisition

**Table S3. Follow-up degree of physical activity**

| Minutes of moderate to vigorous physical activity | Oxiracetam  (N=226) | Placebo  (N=231) | Total  (N=457) | *P*-value^*^ |
| --- | --- | --- | --- | --- |
| Baseline | 23.2$\pm$23.0 | 24.1$\pm$28.7 | 23.7$\pm$26.1 | 0.75 |
| Follow-up #1 | 26.1$\pm$40.4 | 25.0$\pm$31.7 | 25.5$\pm$36.2 | 0.76 |
| Follow-up #2 | 26.1$\pm$33.3 | 25.1$\pm$34.1 | 25.6$\pm$33.7 | 0.79 |
| Follow-up #3 | 22.8$\pm$23.0 | 24.7$\pm$33.2 | 23.8$\pm$28.8 | 0.53 |
| ^*^ p-value by Pearson’s chi-square test | | | | |

**Table S4. Mean changes of primary and secondary outcomes in oxiracetam and placebo groups over 36 weeks**

| Efficacy endpoints | Oxiracetam  (N=226) | | | Placebo  (N=231) | | |
| --- | --- | --- | --- | --- | --- | --- |
|  | Baseline | 36-weeks | *P*^*^ | Baseline | 36-weeks | *P*^*^ |
| MMSE | 26.58$\pm$2.57 | 26.71$\pm$2.71 | 0.051 | 26.64$\pm$2.60 | 26.91$\pm$2.84 | 0.49 |
| CDR-SB | 1.01$\pm$0.81 | 0.87$\pm$0.83 | <0.01 | 0.97$\pm$0.82 | 0.89$\pm$0.99 | 0.15 |
| SVLT-E immediate recall z-score | -0.93$\pm$0.92 | -0.64$\pm$1.05 | <0.01 | -0.89$\pm$0.99 | -0.60$\pm$1.08 | <0.01 |
| SVLT-E delayed recall z-score | -0.88$\pm$1.02 | -0.59$\pm$1.08 | <0.01 | -0.78$\pm$0.98 | -0.60$\pm$1.10 | <0.01 |
| SVLT-E recognition z-score | -0.55$\pm$1.28 | -0.35$\pm$1.17 | 0.02 | -0.63$\pm$1.25 | -0.39$\pm$1.24 | <0.01 |
| Semantic fluency z-score | -0.85$\pm$0.92 | -0.84$\pm$0.89 | 0.78 | -0.80$\pm$0.98 | -0.77$\pm$0.94 | 0.57 |
| Phonemic fluency z-score | -0.90$\pm$0.88 | -0.76$\pm$0.98 | <0.01 | -0.80$\pm$0.93 | -0.63$\pm$0.96 | <0.01 |
| Digit symbol coding z-score | -0.56$\pm$1.07 | -0.48$\pm$1.14 | 0.06 | -0.51$\pm$1.06 | -0.45$\pm$1.10 | 0.052 |
| TMT-E part A z-score | -0.46$\pm$1.28 | -0.46$\pm$1.35 | 0.96 | -0.51$\pm$1.33 | -0.35$\pm$1.29 | 0.03 |
| TMT-E part B z-score | -0.69$\pm$1.77 | -0.56$\pm$1.62 | 0.25 | -0.51$\pm$1.61 | -0.54$\pm$1.92 | 0.70 |
| Short BNT z-score | 0.20$\pm$1.25 | 0.42$\pm$1.04 | <0.01 | 0.10$\pm$1.20 | 0.24$\pm$1.23 | <0.01 |
| RCFT z-score | -0.69$\pm$1.47 | -0.54$\pm$1.51 | 0.07 | -0.57$\pm$1.44 | -0.58$\pm$1.47 | 0.96 |
| NPI-Q | 2.40$\pm$3.51 | 1.83$\pm$3.84 | 0.04 | 1.86$\pm$3.14 | 1.39$\pm$2.94 | 0.03 |
| SGDS | 5.50$\pm$4.30 | 4.59$\pm$4.42 | <0.01 | 4.94$\pm$3.91 | 3.94$\pm$3.91 | <0.01 |
| SGDS $\geq$ 8 | 67 (29.6) | 61 (2.72) |  | 57 (24.7) | 49 (21.2) |  |
| IADL | 0.17$\pm$0.23 | 0.16$\pm$0.28 | 0.52 | 0.17$\pm$0.22 | 0.14$\pm$0.23 | 0.02 |
| IADL $\geq$ 0.43 | 24 (11.3) | 19 (8.9) |  | 22 (10.4) | 18 (9.5) |  |
| Memory domain z-score | -0.78$\pm$0.88 | -0.53$\pm$0.96 | <0.01 | -0.76$\pm$0.87 | -0.53$\pm$0.97 | <0.01 |
| Frontal domain z-score | -0.67$\pm$0.89 | -0.62$\pm$0.91 | 0.06 | -0.63$\pm$0.87 | -0.55$\pm$0.91 | 0.01 |
| Global cognitive function z-score | -0.49$\pm$0.74 | -0.32$\pm$0.79 | <0.01 | -0.46$\pm$0.79 | -0.35$\pm$0.82 | <0.01 |
| EuroQoL | 6.62$\pm$1.54 | 6.38$\pm$1.60 | <0.01 | 6.60$\pm$1.54 | 6.38$\pm$1.66 | 0.02 |
| Global efficiency | 0.85$\pm$0.03 | 0.85$\pm$0.03 | 0.15 | 0.85$\pm$0.03 | 0.85$\pm$0.03 | 0.56 |
| Characteristic path length | 1.26$\pm$0.07 | 1.27$\pm$0.07 | 0.38 | 1.27$\pm$0.07 | 1.27$\pm$0.08 | 0.66 |
| Clustering coefficient | 1.73$\pm$0.28 | 1.76$\pm$0.28 | 0.11 | 1.74$\pm$0.27 | 1.76$\pm$0.30 | 0.51 |
| Modularity | 3.35$\pm$0.41 | 3.38$\pm$0.38 | 0.23 | 3.36$\pm$0.39 | 3.38$\pm$0.43 | 0.64 |

Numbers denote mean ± standard deviations for continuous variables or frequencies (proportions) for categorical variables.

^*^ *P*-value for mean change from baseline to visit 5 by paired t-test

Abbreviations: MMSE = mini-mental state examination; CDR-SB = clinical dementia rating - sum of boxes; SVLT-E = Seoul verbal learning test – elderly version; TMT-E = trail-making test – elderly version; BNT = Boston naming test; RCFT = Rey complex figure copy test; NPI-Q = neuropsychiatric inventory – questionnaire; SGDS = short version of geriatric depression scale; IADL = instrumental activities of daily living; PGA = Patient’s global assessment

**Table S5. Multivariable analysis of efficacy endpoints using linear mixed models adjusting significant covariate differences between groups in modified ITT population**

|  | Coefficients | SE | *LMM*  *P*-value^*^ |
| --- | --- | --- | --- |
| **Primary endpoints** |  |  |  |
| MMSE^†^ | -0.14 | 0.21 | 0.50 |
| CDR-SB^†^ | -0.06 | 0.07 | 0.41 |
| **Secondary endpoints** |  |  |  |
| SVLT-E immediate recall z-score | 0.001 | 0.08 | 0.99 |
| SVLT-E delayed recall z-score | 0.12 | 0.08 | 0.13 |
| SVLT-E recognition z-score | -0.11 | 0.11 | 0.32 |
| Semantic fluency z-score | 0.001 | 0.07 | 0.99 |
| Phonemic fluency z-score | -0.004 | 0.07 | 0.95 |
| Digit symbol coding z-score | 0.02 | 0.052 | 0.65 |
| TMT-E part A z-score | -0.09 | 0.10 | 0.37 |
| TMT-E part B z-score | 0.14 | 0.13 | 0.30 |
| Short BNT z-score | 0.07 | 0.09 | 0.43 |
| RCFT z-score | 0.19 | 0.12 | 0.10 |
| NPI-Q | -0.002 | 0.31 | 0.99 |
| SGDS | 0.21 | 0.31 | 0.50 |
| IADL | 0.02 | 0.02 | 0.27 |
| Memory domain z-score | 0.003 | 0.06 | 0.97 |
| Frontal domain z-score | 0.01 | 0.050 | 0.79 |
| Global cognitive function z-score | 0.07 | 0.04 | 0.14 |
| EuroQoL | -0.001 | 0.12 | 0.995 |
| PGA at Visit 5 | 0.049 | 0.08 | 0.53 |
| Significantly improved |  |  |  |
| Moderately improved |  |  |  |
| Mildly improved |  |  |  |
| Almost the same |  |  |  |
| Mildly worse |  |  |  |
| Moderately worse |  |  |  |
| Significantly worse |  |  |  |
| Global efficiency^‡^ | -0.0003 | 0.004 | 0.94 |
| Characteristic path length^‡^ | -0.002 | 0.01 | 0.89 |
| Clustering coefficient^‡^ | 0.09 | 0.04 | 0.04 |
| Modularity^‡^ | 0.003 | 0.049 | 0.96 |

*^*^ P*-value by linear mixed model treating center effect as random using independence variance-covariance structure

^†^ Change = visit 5 (week 36) – baseline value

^‡^ Normalized values, thresholded at 0.20

Abbreviations: MMSE, mini-mental state examination; CDR-SB, clinical dementia rating - sum of boxes; SVLT-E, Seoul verbal learning test – elderly version; TMT-E, trail-making test – elderly version; BNT, Boston naming test; RCFT, Rey complex figure copy test; NPI-Q, neuropsychiatric inventory – questionnaire; SGDS, short version of geriatric depression scale; IADL, instrumental activities of daily living; PGA, Patient’s global assessment

**Table S6. Sensitivity analysis for efficacy endpoints using sequential condition mean model to account the time-dependent confounder effects of the participants’ degree of physical activity during the trial between groups in modified ITT population**

| Efficacy endpoints at visit 5 | Oxiracetam (N=226) | | | Placebo (N=231) | | | *SCMM*  *P*-value | *Interaction*  *P*-value |
| --- | --- | --- | --- | --- | --- | --- | --- | --- |
|  | baseline | Visit 5 | change | baseline | Visit 5 | change |  |  |
| **Primary endpoints** |  |  |  |  |  |  |  |  |
| MMSE raw score | 26.58$\pm$2.57 | 26.71$\pm$2.72 | 0.13$\pm$2.27 | 26.64$\pm$2.60 | 26.91$\pm$2.85 | 0.27$\pm$2.09 | 0.43 | 0.07 |
| CDR-SB raw score | $1.01\pm$0.81 | 0.87$\pm$0.84 | -0.14$\pm$0.70 | 0.97$\pm$0.82 | 0.89$\pm$0.99 | -0.08$\pm$0.80 | 0.51 | 0.34 |
| **Secondary endpoints** |  |  |  |  |  |  |  |  |
| SVLT-E immediate recall z-score | -0.93$\pm$0.92 | -0.64$\pm$1.05 | 0.28$\pm$0.85 | -0.89$\pm$0.99 | -0.60$\pm$1.08 | 0.28$\pm$0.80 | 0.95 | 0.09 |
| SVLT-E delayed recall z-score | -0.88$\pm$1.02 | -0.59$\pm$1.08 | 0.28$\pm$0.87 | -0.78$\pm$0.98 | -0.60$\pm$1.10 | 0.23$\pm$0.85 | 0.47 | 0.46 |
| SVLT-E recognition z-score | -0.55$\pm$1.28 | -0.35$\pm$1.17 | 0.18$\pm$1.17 | -0.63$\pm$1.25 | -0.39$\pm$1.24 | 0.21$\pm$1.17 | 0.92 | 0.24 |
| Semantic fluency z-score | -0.85$\pm$0.92 | -0.84$\pm$0.89 | 0.01$\pm$0.78 | -0.8$\pm$0.98 | -0.77$\pm$0.94 | 0.03$\pm$0.75 | 0.46 | 0.98 |
| Phonemic fluency z-score | -0.90$\pm$0.88 | -0.76$\pm$0.98 | 0.15$\pm$0.69 | -0.80$\pm0.93$ | -0.63$\pm$0.96 | 0.17$\pm$0.71 | 0.63 | 0.55 |
| Digit symbol coding z-score | -0.56$\pm$1.07 | -0.48$\pm$1.14 | 0.08$\pm$0.60 | -0.51$\pm$1.06 | -0.45$\pm$1.11 | 0.06$\pm$0.50 | 0.57 | 0.11 |
| TMT-E part A z-score | -0.46$\pm$1.28 | -0.46$\pm$1.35 | 0.00$\pm$1.13 | -0.51$\pm$1.33 | -0.36$\pm$1.29 | 0.16$\pm$1.11 | 0.13 | 0.11 |
| TMT-E part B z-score | -0.69$\pm$1.77 | -0.56$\pm$1.62 | 0.12$\pm$1.59 | -0.51$\pm$1.61 | -0.54$\pm$1.92 | -0.03$\pm$1.24 | 0.30 | 0.46 |
| Short BNT z-score | 0.20$\pm$1.25 | 0.42$\pm$1.04 | 0.23$\pm$1.10 | 0.10$\pm$1.20 | 0.24$\pm$1.23 | 0.14$\pm$0.74 | 0.07 | 0.60 |
| RCFT z-score | -0.69$\pm$1.47 | -0.54$\pm$1.51 | 0.15$\pm$1.22 | -0.57$\pm$1.44 | -0.58$\pm$1.47 | 0.00$\pm$1.13 | 0.09 | 0.052 |
| NPI-Q | 2.40$\pm$3.51 | 1.83$\pm$3.84 | -0.49$\pm$3.31 | 1.86$\pm$3.14 | 1.39$\pm$2.94 | -0.46$\pm$2.97 | 0.71 | 0.64 |
| SGDS | 5.50$\pm$4.30 | 4.59$\pm$4.42 | -0.88$\pm$3.46 | 4.94$\pm$3.91 | 3.94$\pm$3.91 | -1.00$\pm$2.88 | 0.51 | 0.20 |
| IADL | 0.17$\pm$0.23 | 0.16$\pm$0.28 | -0.01$\pm$0.20 | 0.17$\pm$0.22 | 0.14$\pm$0.23 | -0.03$\pm$0.18 | 0.43 | 0.84 |
| Memory domain z-score | -0.78$\pm$0.88 | -0.53$\pm$0.96 | 0.246$\pm$0.705 | -0.76$\pm$0.87 | -0.53$\pm$0.97 | 0.234$\pm$0.656 | 0.73 | 0.63 |
| Frontal domain z-score | -0.69$\pm$0.89 | -0.62$\pm$0.91 | 0.073$\pm$0.581 | -0.63$\pm$0.87 | -0.55$\pm$0.91 | 0.078$\pm$0.456 | 0.86 | 0.19 |
| Global cognitive function z-score | -0.49$\pm$0.74 | -0.32$\pm$0.79 | 0.175$\pm$0.514 | -0.46$\pm$0.79 | -0.35$\pm$0.82 | 0.114$\pm$0.389 | 0.04 | 0.22 |
| EuroQoL | 6.62$\pm$1.54 | 6.38$\pm$1.60 | -0.24$\pm$1.13 | 6.60$\pm$1.54 | 6.38$\pm$1.66 | -0.22$\pm$1.41 | 0.90 | 0.17 |
| PGA at Visit 5 |  |  |  |  |  |  | - | - |
| Significantly improved |  | 5 (2.2) |  |  | 5 (2.2) |  |  |  |
| Moderately improved |  | 14 (6.2) |  |  | 22 (9.5) |  |  |  |
| Mildly improved |  | 64 (28.3) |  |  | 64 (27.7) |  |  |  |
| Almost the same |  | 128 (56.6) |  |  | 127 (55.0) |  |  |  |
| Mildly worse |  | 13. (5.8) |  |  | 11 (4.8) |  |  |  |
| Moderately worse |  | 2 (0.9) |  |  | 2 (0.9) |  |  |  |
| Significantly worse |  | 0 (0) |  |  | 0 (0) |  |  |  |
| Global efficiency | 0.85$\pm$0.03 | 0.85$\pm$0.03 | 0.00$\pm$0.04 | 0.85$\pm$0.03 | 0.85$\pm$0.03 | 0.00$\pm$0.04 | 0.52 | 0.70 |
| Characteristic path length | 1.26$\pm$0.07 | 1.27$\pm$0.07 | 0.01$\pm$0.09 | 1.27$\pm$0.07 | 1.27$\pm$0.08 | 0.00$\pm$0.08 | 0.36 | 0.81 |
| Clustering coefficient | 1.73$\pm$0.28 | 1.76$\pm$0.28 | 0.04$\pm$0.33 | 1.74$\pm$0.27 | 1.76$\pm$0.30 | 0.02$\pm$0.34 | 0.20 | 0.96 |
| Modularity | 3.35$\pm$0.41 | 3.38$\pm$0.38 | 0.04$\pm$0.48 | 3.36$\pm$0.39 | 3.38$\pm$0.43 | 0.02$\pm$0.52 | 0.76 | 0.73 |

Numbers denote mean ± standard deviations for continuous variables or frequencies (proportions) for categorical variables.

Abbreviations: ITT, intention-to-treat; SCMM, sequential condition mean model; MMSE, mini-mental state examination; CDR-SB, clinical dementia rating - sum of boxes; SVLT-E, Seoul verbal learning test – elderly version; TMT-E, trail-making test – elderly version; BNT, Boston naming test; RCFT, Rey complex figure copy test; NPI-Q, neuropsychiatric inventory – questionnaire; SGDS, short version of geriatric depression scale; IADL, instrumental activities of daily living; PGA, Patient’s global assessment

**Figure S1. Distribution of MMSE score changes from baseline to week 36 in the oxiracetam and placebo groups**


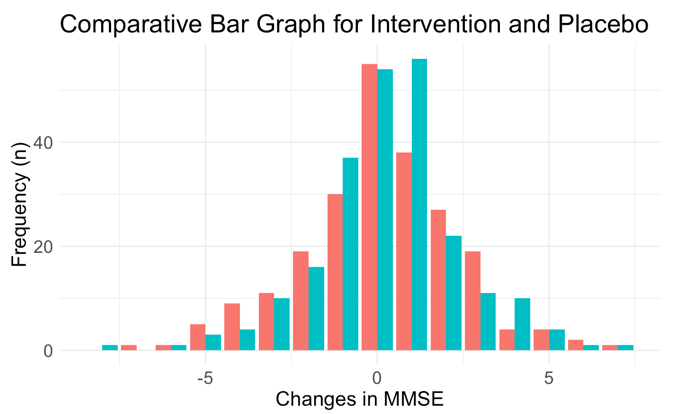

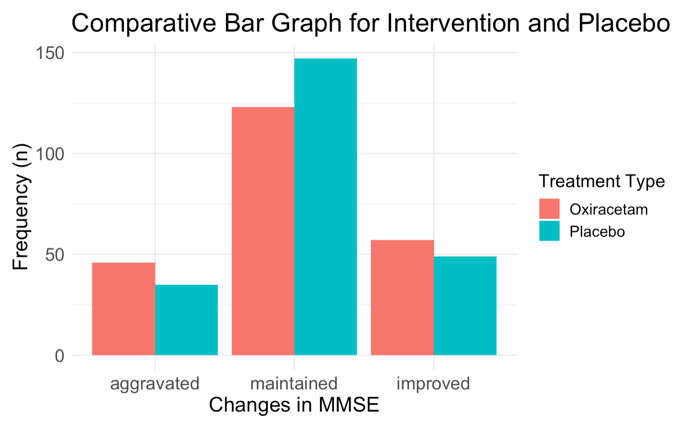


Cognitive outcome categories were defined as follows: “Improved” indicates an increase of 2 or more points in MMSE score; “Aggravated” indicates a decrease of 2 or more points; and “Maintained” refers to changes within ±1 point from baseline.

Abbreviations: MMSE = mini-mental state examination

**Figure S2. Changes in MMSE and CDR-SB according to physical activity tertiles**

The CDR-SB, like the MMSE, was inverted and plotted on the graph to reflect improving condition as the line goes up. As physical activity at baseline was balanced across randomized groups, the mean changes in MMSE and CDR-SB scores (follow-up [V5] - baseline scores [V1]) between treatment groups were compared using ANOVA test. In addition, the significance of the interaction effect between treatment group and baseline physical activity was tested using a 5% significance level through multiple linear regression analysis with sequential conditional mean model (SCMM).

**
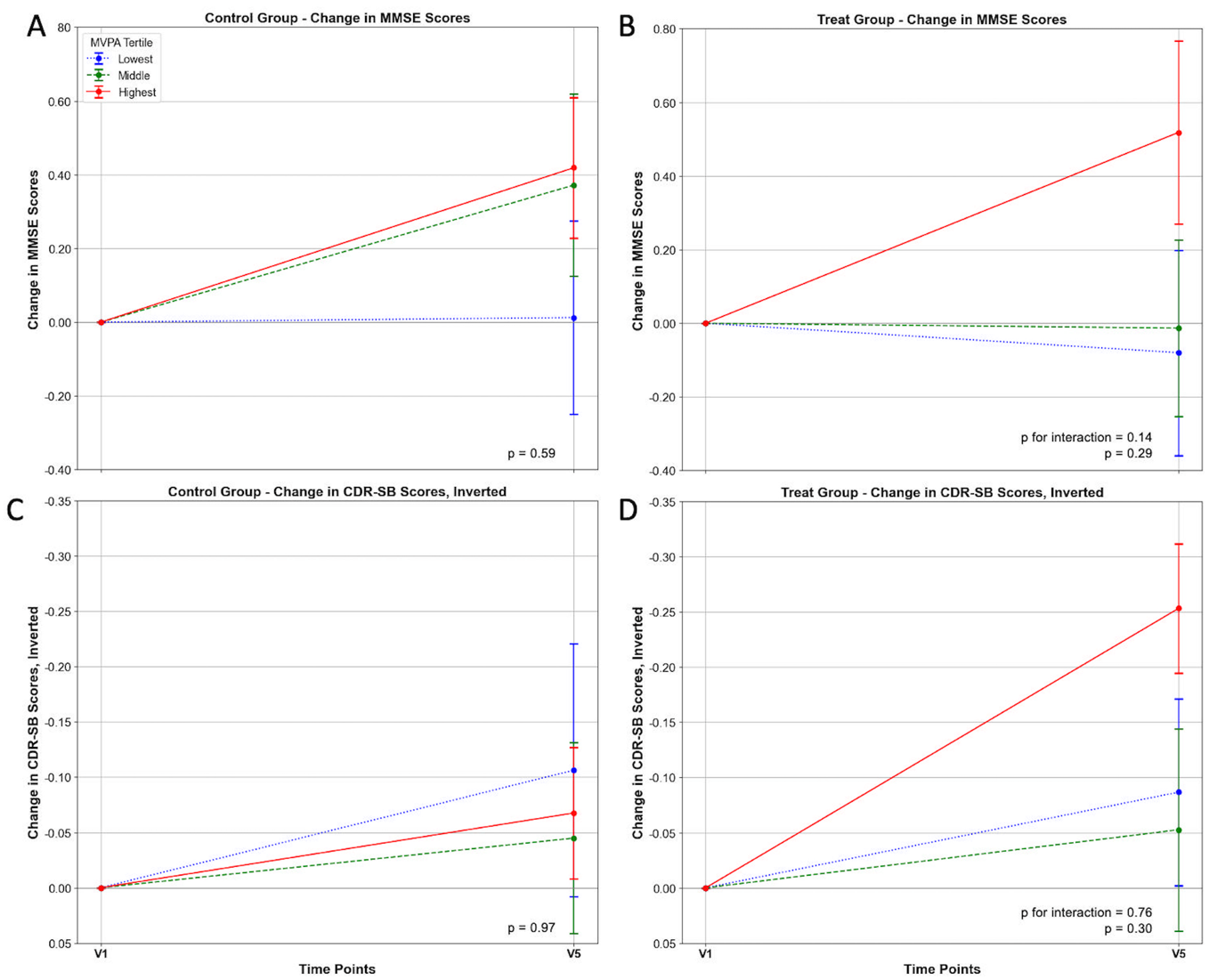
**

**Supplemental References**

1. Kang Y, Chin J, Na DL, et al. A normative study of the Korean version of Controlled Oral Word Association Test (COWAT) in the elderly. *Korean J Clin Psychol* 2000; 19: 385–392.

2. Yum T, Park Y, Oh-hashi K, et al. *Manual for Korean-Wechsler Adult Intelligence Scale*. Seoul: Korea Guidance, 1992.

3. Lee HS, Chin J, Lee BH, et al. Development and validation of Korean version of trail making test for elderly persons. *Dement Neurocognitive Disord* 2007; 6: 54–66.

4. Kang Y, Kim H, Na DL. Parallel Short Forms for the Korean-Boston Naming Test (K-BNT). *J Korean Neurol Assoc* 2000; 18: 144–150.

5. Kang Y, Na DL. N. *Professional manual; Seoul neuropsychological screening battery.* Seoul: Human brain research and consulting, 2003.

6. Keogh RH, Daniel RM, VanderWeele TJ, et al. Analysis of Longitudinal Studies With Repeated Outcome Measures: Adjusting for Time-Dependent Confounding Using Conventional Methods. *Am J Epidemiol* 2017; 187: 1085–1092.
